# Antiviral Efficacy of the Anesthetic Propofol against Dengue Virus Infection and Cellular Inflammation

**DOI:** 10.1155/2021/6680913

**Published:** 2021-03-31

**Authors:** Ting-Jing Shen, Chia-Ling Chen, Ming-Kai Jhan, Po-Chun Tseng, Rahmat Dani Satria, Chung-Hsi Hsing, Chiou-Feng Lin

**Affiliations:** ^1^Graduate Institute of Medical Sciences, College of Medicine, Taipei Medical University, Taipei 110, Taiwan; ^2^Department of Microbiology and Immunology, School of Medicine, College of Medicine, Taipei Medical University, Taipei 110, Taiwan; ^3^School of Respiratory Therapy, College of Medicine, Taipei Medical University, Taipei 110, Taiwan; ^4^Core Laboratory of Immune Monitoring, Office of Research & Development, Taipei Medical University, Taipei 110, Taiwan; ^5^International Ph.D. Program in Medicine, College of Medicine, Taipei Medical University, Taipei 110, Taiwan; ^6^Department of Clinical Pathology and Laboratory Medicine, Faculty of Medicine, Public Health and Nursing, Universitas Gadjah Mada, Yogyakarta 55281, Indonesia; ^7^Clinical Laboratory Installation, Dr. Sardjito Central General Hospital, Yogyakarta 55281, Indonesia; ^8^Department of Medical Research, Chi-Mei Medical Center, Tainan 710, Taiwan; ^9^Department of Anesthesiology, Chi-Mei Medical Center, Tainan 710, Taiwan; ^10^Center of Infectious Diseases and Signaling Research, National Cheng Kung University, Tainan 701, Taiwan

## Abstract

Propofol, 2,6-diisopropylphenol, is a short-acting intravenous sedative agent used in adults and children. Current studies show its various antimicrobial as well as anti-inflammatory effects. Dengue virus (DENV) is an emerging infectious pathogen transmitted by mosquitoes that causes mild dengue fever and progressive severe dengue diseases. In the absence of safe vaccines and antiviral agents, adjuvant treatments and supportive care are generally administered. This study investigated the antiviral effects of propofol against DENV infection and cellular inflammation by using an *in vitro* cell model. Treatment with propofol significantly inhibited DENV release 24 h postinfection in BHK-21 cells. Furthermore, it also blocked viral protein expression independent of the translational blockade. Propofol neither caused inhibitory effects on endosomal acidification nor prevented dsRNA replication. Either the proinflammatory TNF-*α* or the antiviral STAT1 signaling was reduced by propofol treatment. These results provide evidence to show the potential antiviral effects of the sedative propofol against DENV infection and cellular inflammation.

## 1. Introduction

The anesthetic propofol is routinely used in the short term to provide a rapid onset and offset of sedation in critically ill patients under intensive care [1]. Treatment with propofol confers a range of pharmacodynamic effects from amnestic, muscle relaxant, and hypnotic effects to anesthesia. In addition to its neuropharmacological properties, propofol has immunomodulating actions through its negative regulation of proinflammatory cytokine/chemokine production and immune cell activation [2, 3]. Additionally, general anesthetics, including propofol, remifentanil, and ketamine, exert antimicrobial and microbial growth-promoting effects against bacterial infection [4, 5].

Dengue, an arthropod-borne viral disease, is caused by the dengue virus (DENV), a flavivirus transmitted by Aedes mosquitoes [6]. Globally, infection with DENV affects more than 100 countries, with an estimated 400 million new infections and 25,000 deaths annually [7]. Clinical presentations of DENV infection range from mild dengue fever to severe dengue diseases, including dengue hemorrhagic fever/dengue shock syndrome (DHF/DSS) and multiorgan involvement. Without appropriate medication, severe dengue has a mortality rate ranging from 5 to 20%. Due to its emerging disease status, safe and long-term protective DENV vaccines and anti-DENV drugs are essential for dengue prevention and treatment.

For severe dengue management, some sedative agents are used in patients [8, 9]. An innovative patent describes the application of propofol as an antiviral medication for preventing and treating infections caused by influenza A viruses (EP2590637A1; European Patent Office). However, its pharmacological effects on viral replication as well as viral inflammation remain undefined. In this study, we first investigated the possible effects of propofol on DENV infection and replication and DENV-induced cellular inflammation by using an *in vitro* cell model.

## 2. Materials and Methods

### 2.1. Cells and Virus

The process of cell culture and virus preparation was according to our previous study [10]. Briefly, baby hamster kidney- (BHK-) 21 fibroblasts (ATCC, CCL10) were maintained in Dulbecco's modified Eagle's medium (DMEM; Thermo Fisher Scientific) containing 10% heat-inactivated fetal bovine serum (FBS, Biological Industries) and 1% penicillin-streptomycin (Thermo Fisher Scientific) at 37°C in 5% CO_2_. BHK-21 cells harboring a luciferase-expressing DENV replicon (BHK-D2-Fluc-SGR-Neo-1) were maintained in DMEM with 10% heat-inactivated FBS, 1% penicillin-streptomycin, and 0.4 mg/ml G418 agent (Cat# A1720, Sigma-Aldrich) at 37°C in 5% CO_2_. The *Aedes albopictus* clone mosquito C6/36 cells (ATCC, CRL1660) were maintained in Minimum Essential Medium (MEM; Thermo Fisher Scientific) containing 10% heat-inactivated FBS, 1% penicillin-streptomycin (Thermo Fisher Scientific), 1% sodium pyruvate (Cat# 11360-070, Thermo Fisher Scientific), 1% 4-(2-hydroxyethyl)-1-piperazineethanesulfonic acid (HEPES; Cat# 15630-080, Thermo Fisher Scientific), and 1% nonessential amino acids (NEAA; Cat# 11140-035, Thermo Fisher Scientific) at 28°C in 5% CO_2_. Dengue virus serotype 2 (DENV2, strain PL046) was obtained from Center for Disease Control in Taiwan and propagated in a C6/36 cell monolayer at a multiplicity of infection (MOI) of 0.01. After incubation (28°C in 5% CO_2_) for 5 days, the viral supernatants were collected and filtered with a 0.22 *μ*m filter and then stored at -80°C until use. Viral titers were determined by plaque assay using BHK-21 cells.

### 2.2. Agents and Antibodies

Propofol (2,6-diisopropylphenol) was purchased from Sigma-Aldrich (St. Louis, MO, USA). An antibody against DENV NS1 (Cat# GTX124280) was purchased from GeneTex (San Antonio, TX); antibodies against phospho-STAT1^Tyr701^ (Cat# 9167; clone 58D6), STAT1 (Cat# 9172), horseradish peroxidase- (HRP-) conjugated goat anti-rabbit IgG (Cat# 7074S), and HRP-conjugated horse anti-mouse IgG (Cat# 7076S) were purchased from Cell Signaling Technology (Beverly, MA); Alexa Flour 488-conjugated goat anti-mouse antibody (Cat# A-11029) and Hoechst 33258 (Cat# H3569) were purchased from Thermo Fisher Scientific (Pittsburgh, PA, USA); antibody against dsRNA (Cat# 10010200) was purchased from SCICONS; antibody against mouse *β*-actin (Cat# A5441), 4,6-diamidino-2-phenylindole (DAPI; Cat# D9542), the V-ATPase inhibitor bafilomycin A1 (Baf A1; Cat# 19-148), and acridine orange hemi (zinc chloride) salt (Cat# A6014) were purchased from Sigma-Aldrich (St. Louis, MO). According to the manufacturer's instructions, cell cytotoxicity was assessed using Cytotoxicity Detection Kit assays (Roche Diagnostics, Lewes, UK).

### 2.3. Western Blotting

Accordingly [10], cells were collected and extracted with lysis buffer containing a protease inhibitor cocktail (Sigma-Aldrich). The processed proteins were separated by 10% SDS-polyacrylamide gel electrophoresis followed by transfer to a polyvinylidene difluoride (PVDF) membrane (Millipore). Then, the PVDF membrane was blocked with 5% nonfat milk in 0.05% Tween-20-containing Tris-buffer-based saline (TBS-T) at room temperature for 1 h. Next, the membrane was washed three times with TBS-T buffer and immunohybridized with the indicated primary antibodies at 4°C overnight. Then, the membrane was washed with TBS-T buffer three times, followed by incubation with the indicated HRP-conjugated secondary antibodies at room temperature for 1 h. The antibody-protein complexes on the PVDF membrane were detected using an ECL Western blot detection kit (PerkinElmer). The signals of the identified proteins were captured with a film exposure system.

### 2.4. Plaque Assay

BHK-21 cells were grown in a monolayer in a 12-well plate at 7 × 10^4^ cells/well. Serially diluted viral solutions were added to infect cells for 2 h and then replaced with fresh DMEM containing 4% FBS and 0.5% methylcellulose (Sigma-Aldrich) for 5 days. Next, wells were washed with 2 ml PBS twice and stained with crystal violet solution containing 1% crystal violet (Sigma-Aldrich), 0.64% NaCl, and 2% paraformaldehyde (Sigma-Aldrich) overnight. Subsequently, wells were washed with water and air-dried to count the number of plaque-forming units (PFU).

### 2.5. Reporter Assay

BHK-D2-Fluc-SGR-Neo-1 cells (replicons) were seeded in 96-well plates at 3,000 cells/well overnight. After the treatments, luciferase activity was detected using the Dual-Glo® Luciferase Assay System (Cat# E2940, Promega) and a spectral scanning multimode reader (Thermo Varioskan Flash).

### 2.6. Double-stranded RNA (dsRNA) Staining

Cells were washed with ice-cold PBS 3 times and fixed with 4% paraformaldehyde (Sigma-Aldrich) at room temperature for 15 minutes. Then, the cells were washed 3 times with ice-cold PBS and permeabilized with permeabilization buffer (PBS containing 1% Triton X-100) at room temperature for 5 minutes. The cells were then washed 3 times with ice-cold PBS and immunoblocked with blocking buffer (PBS containing 1% BSA and 0.01% Triton X-100) at 4°C for 30 minutes. Next, the cells were washed 3 times with ice-cold PBS and immunohybridized with mouse anti-dsRNA J2 primary antibody at 4°C overnight. Subsequently, the cells were washed 3 times with ice-cold PBS and stained with Alexa Fluor 488-conjugated goat anti-mouse antibody (Thermo Fisher Scientific) at room temperature for 15 minutes. The cells were washed 3 times with ice-cold PBS and then visualized with fluorescence or confocal microscopy. DAPI (Sigma-Aldrich) was used for nuclear staining.

### 2.7. Acridine Orange Staining

Cells were washed with HBSS (Thermo Fisher Scientific) once and then stained with acridine orange agent (Sigma-Aldrich) and Hoechst 33258 (Thermo Fisher Scientific) in an incubator at 37°C in 5% CO_2_. After 45 minutes, the cells were washed with HBSS once and rinsed with HBSS. Subsequently, cells were visualized with a fluorescence microscope (EVOS). Hoechst 33258 was used for nuclear staining.

### 2.8. Enzyme-Linked Immunosorbent Assay (ELISA)

According to the manufacturer's instruction, samples were harvested, and the concentration of mouse TNF*α* was determined using ELISA kit (Cat# 88-7324-88, eBioscience).

### 2.9. Statistical Analysis

GraphPad Prism (version 8.3.0) was applied to analyze the experimental data. One-way ANOVA (Tukey's multiple comparison test) was used to determine experiments involving numerous groups. Values are means ± standard deviation (SD). All *p* values were obtained from two-tailed significance tests. A *p* value of <0.05 was considered statistically significant.

## 3. Results

### 3.1. Propofol Treatment Inhibits DENV Infection

**Figure 1:**
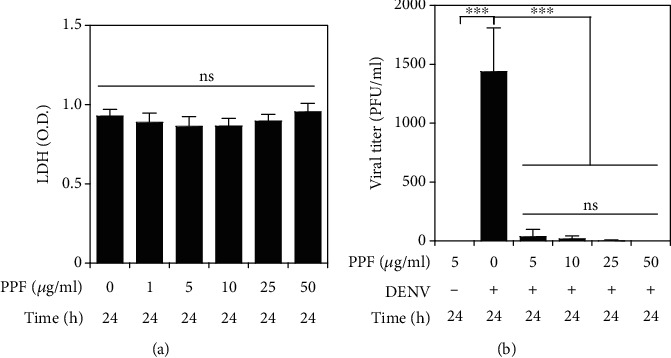
Propofol treatment inhibits DENV virion production. (a) The LDH assay showed the cytotoxicity of BHK-21 cells cultured in a medium containing propofol for 24 h. (b) Plaque assay determined the viral titer in BHK-21 cells pretreated with propofol for 1 h followed by DENV2 (MOI = 1) infection for 24 h. Quantitative data are presented as the mean ± SD of at least three independent experiments (*n* = 3). ^∗∗∗^*p* < 0.001. ns: not significant.

Several antiviral drugs have been designed and repurposed to reduce DENV infection [11]. Here, we examined propofol, a short-term anesthetic, for its antiviral activity. The LDH assay showed that propofol did not cause cytotoxicity at testing dosages ranging from 1 to 50 *μ*g/ml (Figure 1(a)). Based on these results, BHK-21 cells were pretreated with propofol for 1 h and then infected with DENV for an additional 24 h. In this DENV infection model, 24 h postinfection showed a significant viral replication as demonstrated by using plaque assay. The results showed that propofol significantly (*p* < 0.001) reduced DENV virion release, as demonstrated by the viral titer, at doses of 5, 10, 25, and 50 *μ*g/ml (Figure 1(b)). These results indicate that propofol treatment effectively blocks DENV infection.

### 3.2. Propofol Reduces DENV Viral Protein Expression but Does Not Affect Viral Translation

**Figure 2:**
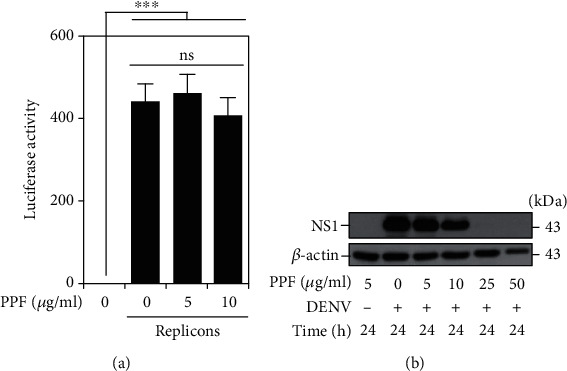
Propofol treatment reduces DENV viral NS1 protein expression but does not affect viral protein translation. (a) Luciferase activities of BHK-21 cells and replicons treated with or without propofol were used to determine viral protein translation. (b) Western blot analysis showed viral NS1 protein expression in BHK-21 cells pretreated with propofol for 1 h and then infected with DENV2 (MOI = 1) for 24 h. Quantitative data are presented as the mean ± SD of at least three independent experiments (*n* = 3). ^∗∗∗^*p* < 0.001. ns: not significant.

To assess propofol's inhibitory activity on DENV infection, we next used BHK-21-SGR cells, a cellular replicon-based reporter assay, to examine propofol's translational targets. The luciferase activity showed no remarkable difference in replicons treated with or without propofol (Figure 2(a)), indicating that a protein translation-independent route mediates the propofol-induced antiviral effect. By western blot analysis, we found that viral NS1 protein expression was effectively increased 24 h postinfection and was decreased in a dose-dependent manner under propofol treatment (Figure 2(b)). Overall, propofol inhibits DENV viral protein expression independent of the translational blockade.

### 3.3. Propofol Does Not Affect DENV-Induced Endosomal Acidification

**Figure 3:**
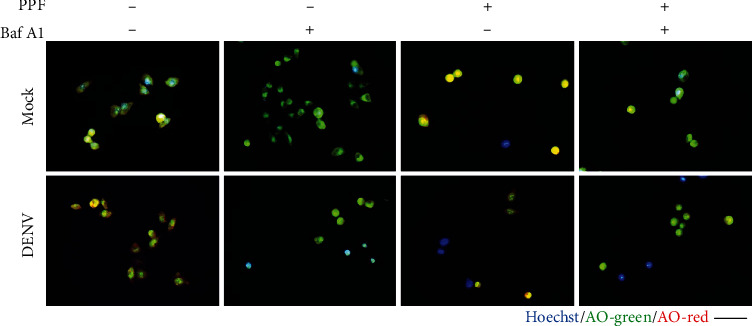
Propofol treatment does not affect endosomal acidification during DENV infection. The fluorescent images of acridine orange-stained BHK-21 cells pretreated with or without Baf A1 and propofol for 1 h showed endosomal acidification during mock and DENV infection for 2 h. Hoechst (blue) was used to label nuclear DNA. Scale bar: 100 *μ*m.

The endosomal acidification step is critical for DENV uncoating to release the viral genome for further replication in the cytoplasm [6]. Therefore, the pH-sensitive dye acridine orange (AO) was used to explore whether propofol affects the early stages of DENV infection accordingly [12]. After a 2-hour infection, images of AO-stained BHK-21 cells showed a low pH in the endosomes (red) of DENV-infected cells compared with mock-infected cells. Cells treated with bafilomycin (Baf) A1, a V-ATPase inhibitor [12], were intensely stained green, indicating that endosome acidification was blocked. Notably, cells treated with propofol exhibited a red color attenuated by cotreatment with Baf A1 (Figure 3). These results reveal that propofol does not affect endosomal acidification during DENV infection.

### 3.4. Propofol Does Not Inhibit dsRNA Replication at the Early Infectious Stage

**Figure 4:**
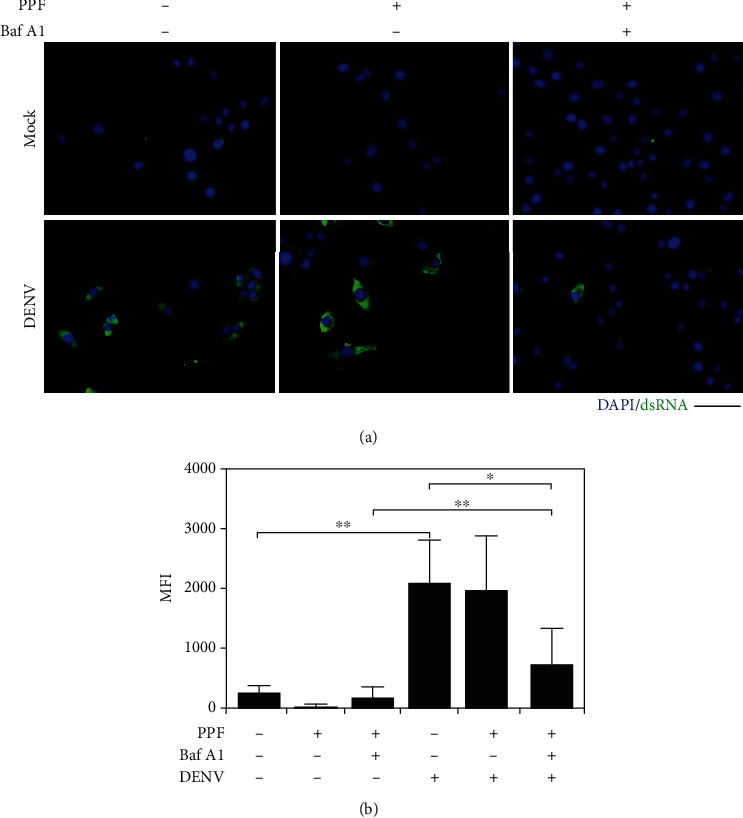
Propofol treatment has no blocking effect on viral dsRNA replication during DENV infection. (a) Images of immunocytochemistry staining showed viral dsRNA (green) expression 6 h postinfection in mock- and DENV2-infected BHK-21 cells pretreated with or without Baf A1 and propofol for 1 h. DAPI (blue) was used to label nuclear DNA. (b) Statistical analysis of the staining presented as mean fluorescent intensity (MFI). Quantitative data are presented as the mean ± SD of the experiments (*n* = 3). ^∗^*p* < 0.05; ^∗∗^*p* < 0.01. Scale bar: 100 *μ*m.

Following endocytosis, the viral genome is released into the cytoplasm and undergoes genome replication [6]. To investigate propofol's inhibitory effect on viral RNA replication, cells were pretreated with the indicated drugs for 1 h and then infected with DENV for an additional 6 h. Images of dsRNA immunostaining showed that dsRNA expression occurred, as demonstrated by positive staining (green), in both cells treated with and without propofol under DENV infection. However, cells pretreated with Baf A1 as a positive control then treated with propofol showed reduced dsRNA expression in the infected cells (Figures 4(a) and 4(b)). The data demonstrate that propofol does not affect blocking DENV viral dsRNA replication at early infection.

### 3.5. Propofol Impedes Proinflammatory Cytokine Responses

**Figure 5:**
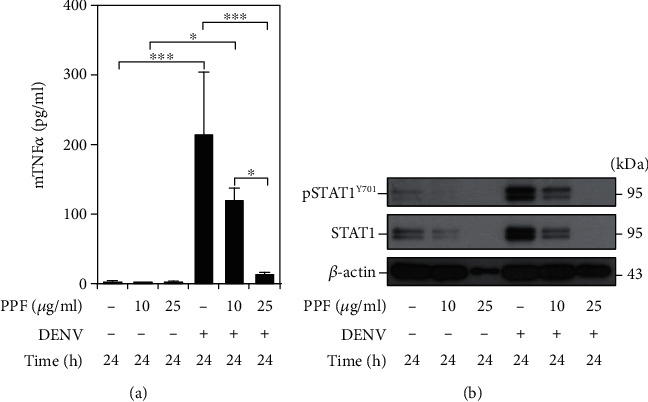
Propofol treatment reduces proinflammatory TNF-*α* production and type I IFN signaling. RAW 264.7 cells were pretreated without or with propofol (10 or 25 *μ*g/ml) followed by infection with DENV2 (MOI = 10) for 24 h. (a) The ELISA analysis determined mouse TNF-*α* production. (b) Western blot analysis showed protein expressions of phospho-STAT1 and STAT1. Quantitative data are presented as the mean ± SD of the experiments (*n* = 3). ^∗^*p* < 0.05; ^∗∗∗^*p* < 0.001.

DENV infection induces robust cytokine productions, such as TNF-*α*, which is immunopathogenic *in vivo* and *in vitro*, to defeat hosts [13–15]. Although propofol is typically used as an anesthetic agent, it also functions to eliminate inflammation. Propofol reduces both the gene and cytokine productions of TNF-*α* in not only CoCl_2_-treated hypoxic BV2 microglia but also cecal ligation and puncture-administrated rat liver [16, 17]. In this study, RAW 264.7 cells were pretreated with or without propofol (10 or 25 *μ*g/ml) followed by infected with DENV for 24 h. ELISA analysis showed that DENV infection significantly increased mouse TNF-*α* production; however, propofol treatment reduced the cytokine levels in a dose-dependent manner (Figure 5(a)). Regarding propofol treatment that inhibits DENV replication, it is hypothesized that propofol may enhance antiviral interferon (IFN) responses. The protein expressions of phospho-STAT1 and STAT1, the expected transcription factor of antiviral IFN signaling, were increased following DENV infection but inhibited by propofol treatment (Figure 5(b)). Thus, it is suggested that propofol has anti-inflammation activity against DENV-induced cellular inflammation.

## 4. Discussion

As a sedative agent, the anesthetic propofol is usually used in critically ill patients [1]. Although it is not a standard medication used in severe dengue patients, its immunomodulatory effects have been reported to inhibit inflammation. Regarding its dual antimicrobial [4] and promicrobial impact [18], this compound confers intense antiviral action against enveloped viruses, especially the influenza viruses (EP2590637A1; European Patent Office). However, propofol is an emulsion of soybean oil, glycerol, and egg lecithin that may prolong the environmental stability to sustain the survival of the hepatitis C virus under this lipid-based formulation [19]. Therefore, the treatment time and the dosage of propofol used for antimicrobial therapy need full consideration. As demonstrated in this *in vitro* cell model, our study is the first to report an antiviral effect of propofol against flavivirus DENV infection.

To date, no licensed antiviral drugs are available for treating DENV. Several potential agents, including chloroquine (a 9-aminoquinoline), balapiravir (4′-azidocytidine), prednisolone, lovastatin, and the or*α*-glucosidase inhibitor celgosivir, are relatively limited by their unusable antiviral effects in dengue patients [20–22]. To speed the development of antiviral agents against DENV infection, repurposing FDA-approved agents is an alternative strategy to target DENV infection and replication [23]. Host factor cyclooxygenase- (COX-) 2 can facilitate DENV replication, and pharmacologically inhibiting its kinase activity has been demonstrated as a potential antiviral strategy [24]. Significantly, treating macrophages with propofol could modulate cellular inflammation via COX activity suppression [25, 26]. It is speculated that the antiviral activity of propofol is mediated by targeting the COX signaling pathway.

Administration of propofol significantly inhibits virion release following the inhibition of viral protein expression; however, propofol treatment does not block DENV-induced endosomal acidification or viral dsRNA replication. Based on our findings, as demonstrated by using a replicon-based reporter system, propofol did not interfere with viral protein translation. All these results indicate a possible effect initiated by propofol targeting protein posttranslational modification. Although a COX-regulated viral infection may act on viral gene transcription, protein expression, and viral genome replication [27], its possible interaction with host factors may also control the posttranslational regulation of viral proteins [28]. Further investigations for exploring propofol-mediated inhibition on the DENV infectious cycle, such as viral protein posttranslational modification, virion assemble, and release, are needed to identify propofol's antiviral actions.

In addition to the modulation of cellular inflammation through suppressing COX activity in macrophages, propofol attenuates neuroinflammation. Peng et al. found that propofol treatment blocks NF-*κ*B/Hif-1*α* signaling and reduces the gene expressions and cytokine secretions of TNF-*α*, IL-1*β*, and IL-6 in hypoxic BV2 microglia [16]. Propofol treatment also could remarkably inhibit gene expressions of TNF-*α*, TLR-4, CD14, and GM-CSF in cultured hepatocytes under LPS stimulation [29]. Besides, propofol exposure causes the reductive numbers of TNF-*α* and inducible nitric oxide synthase-producing dendritic cells in *Listeria monocytogenes*-infected mouse spleens, which therefore reduces the sufficient bacterial clearance [30]. Proinflammatory cytokines such as TNF-*α* are positively associated with severe dengue disease [31]. Jhan et al. further revealed that the blockade of TNF-*α* by knocking the *TNF-α* gene or neutralizing TNF-*α* could reduce the disease severity and mortality in DENV-infected mice [15]. Therefore, in consideration of propofol's inhibition activity on TNF-*α* production, propofol treatment may be a considerable agent to prohibit dengue disease progression.

In conclusion, by using an *in vitro* cell model of DENV infection, for the first time, we demonstrate the antiviral capacity of propofol against DENV infection, which probably occurs through a mechanism involving the blockade of viral protein expression independent of translational inhibition as well as the increase in an antiviral interferon response. While propofol is used as a sedative agent, this study's findings further provide evidence to show the potential antiviral and anti-inflammatory application of propofol in patients with DENV infection. In considering safe dose used in the clinic, an *in vivo* model of DENV infection is essential for evaluating its antiviral and anti-inflammatory effects.

## Data Availability

The data used to support the findings of this study are available from the corresponding author upon request.
